# Combined IFN-γ and TNF-α treatment enhances the susceptibility of breast cancer cells and spheroids to Natural Killer cell-mediated killing

**DOI:** 10.1038/s41419-025-08021-0

**Published:** 2025-10-16

**Authors:** Francesca Barberini, Riccardo Pietroni, Simone Ielpo, Valeria Lucarini, Daniela Nardozi, Ombretta Melaiu, Monica Benvenuto, Chiara Focaccetti, Camilla Palumbo, Federica Rossin, Doriana Fruci, Daniel Olive, Laura Masuelli, Roberto Bei, Loredana Cifaldi

**Affiliations:** 1 Department of Clinical Sciences and Translational Medicine, University of Rome “Tor Vergata”, Rome, Italy; 2 Department of Experimental Medicine, University of Rome “Sapienza”, Rome, Italy; 3 Department of Biology, University of Rome “Tor Vergata”, Rome, Italy; 4 Bambino Gesù Children’s Hospital, IRCCS, Rome, Italy; 5 Immunity and Cancer Team, Cancer Research Center of Marseille, CRCM, INSERM U1068, CNRS UMR7258, Aix-Marseille University U105, Marseille, France

**Keywords:** Breast cancer, Immune cell death, Tumour immunology, NK cells

## Abstract

NK cell-based immunotherapy of solid tumors has been shown to be increasingly successful, but much effort is still needed to optimize its efficacy. This study explores the effects of treatment with low, non-toxic doses of IFN-γ and TNF-α on the susceptibility of breast cancer (BC) cell lines (MCF-7, MDA-MB-231, and MDA-MB-468) cultured in 2D and 3D as spheroids, to NK cell-mediated antitumor function. We evaluated the expression of (i) ligands for NK cell-activating receptors on BC cells, (ii) death and adhesion molecules on BC cells, and (iii) the expression of NK cell-receptors on NK cells infiltrating BC spheroids. Cytokine treatment significantly increased the expression of FAS, TRAIL-R2, and ICAM-1 in all BC cell lines, enhancing NK cell-mediated apoptosis and promoting NK cell-tumor cell conjugate formation. Differently, the expression of ligands for activating receptors remained essentially unchanged. In BC spheroids, the treatment with IFN-γ and TNF-α enhanced NK-cell infiltration, with increased expression of activating receptors (NKG2D, DNAM-1, NKp30, and NKp46) on infiltrating NK cells. However, regardless of treatment, markers of NK cell exhaustion, such as PD-1 and CTLA-4, were also upregulated, the latter especially in triple-negative BC (TNBC) MDA-MB-231 spheroids, thus suggesting an exhausted phenotype of NK cells infiltrating spheroids despite activation. Cytokine treatment resulted in a significant NK cell-mediated reduction in spheroid size, accompanied by an increased apoptotic state, with effects more pronounced in MCF-7 than in MDA-MB-231 spheroids. These results indicate that, although the treatment with IFN-γ and TNF-α improves NK cell-mediated tumor interaction and apoptosis, its therapeutic efficacy may be dependent on the BC subtype, with TNBC spheroids showing greater resistance. These findings highlight the importance of the tumor microenvironment (TME) in shaping NK cell responses and suggest that combining IFN-γ and TNF-α treatments with NK-cell-based immunotherapeutic strategies may improve treatment outcomes, particularly for more aggressive BC subtypes.

## Introduction

Breast cancer (BC) remains one of the most prevalent cancers worldwide, with various subtypes exhibiting distinct clinical behaviors and therapeutic responses [1]. Among these, triple-negative BC (TNBC) is particularly challenging due to its aggressive nature, lack of effective targeted therapies, and poorer prognosis compared to hormone receptor-positive subtypes [2, 3]. Traditional treatment options, including chemotherapy and radiation therapy, have limited efficacy, especially in advanced stages of the disease [4]. Consequently, there is a growing interest in immunotherapeutic approaches, particularly those involving natural killer (NK) cells [5], which are key components of the innate immune system and play an important role in the surveillance and elimination of tumor cells [6].

NK cells exert their antitumor effects through a variety of mechanisms, including the recognition of stress-induced ligands on tumor cells, the release of cytotoxic granules [6], and the induction of tumor cell apoptosis via death receptors such as FAS and TRAIL-R2 [7]. The activation of NK cells is primarily mediated by activating receptors (NKARs), such as NKG2D and DNAM-1, which recognize ligands upregulated on tumor cells in response to cellular stress [8, 9]. It is counterbalanced by the recognition of major histocompatibility complex class I (MHC-I) molecules by killer inhibitory receptors (KIRs), with have a long cytoplasmic tail containing immunoreceptor tyrosine-based inhibition (ITIM) motifs, thus mediating inhibitory signals. Indeed, the expression of MHC-I molecules by healthy cells makes NK cells tolerant toward self and activated against MHC-I -negative or low-expressing tumor cells, as activation signals outweigh inhibitory ones in this last context [10]. Furthermore, the expression of other inhibitory receptors such as NKG2A, immune check point molecules such as PD-1 and CTLA-4, and other activating receptors such as natural cytotoxic receptors (NCRs) and activating KIRs expressing short cytoplasmic tails contribute to NK cell function in the tumor microenvironment (TME) [10]. However, the efficacy of NK cells against solid tumors, including BC, is often compromised by an immunosuppressive TME, which can hinder NK cell activation, infiltration, and function [11]. Therefore, molecular strategies aimed at enhancing NK cell activating signals represent a fundamental approach for strengthening NK cell-based tumor immunotherapy [6].

Cytokines, such as IFN-γ and TNF-α, are potent immunomodulatory molecules that can effectively trigger tumor cell death and exert powerful antitumor effects through multiple mechanisms [12], including the enhancement of NK cell activity [13] and the up-regulation of adhesion molecules on tumor cells [14]. In addition, these cytokines induce the expression of death molecules such as FAS and TRAL-R2 in tumor [15, 16] and non-transformed cells [17–21]. Despite their known effects on immunomodulation, the combined impact of IFN-γ and TNF-α on tumor cell recognition and apoptosis by NK cells, as well as the infiltration of NK cells into the TME, particularly in the context of tumor spheroids, remains poorly explored.

In this study, we investigated the immunomodulatory effects of a combined treatment with low, non-toxic doses of IFN-γ + TNF-α on BC cell lines cultured in two-dimensional (2D) and three-dimensional (3D) spheroids-like patterns, focusing on the expression of ligands for NK cell-activating receptors, death receptors such as FAS and TRAIL-R2, and the adhesion molecule such as ICAM-1. We explored the functional consequences of these changes on NK cell-mediated BC cell apoptosis, the infiltration of NK cells into 3D tumor spheroids, and the immune phenotype of NK cells infiltrating spheroids. Using both 2D and 3D models of BC cells, we unraveled the mechanisms by which treatment with IFN-γ + TNF-α can enhance NK cell function and potentially overcome the immune evasion mechanisms employed by tumors, particularly in aggressive BC subtypes such as TNBC. Our findings provide novel insights into the potential of the cytokines IFN-γ and TNF-α as adjuvants to NK cell-based immunotherapy for the treatment of BC.

## Materials and Methods

### Antibodies and Flow Cytometry

The following antibodies for flow cytometry were used: anti-CD107a-FITC (H4A3), anti-CD3-AlexaFluor-700 (UCHT1), anti-CD56-PE-Cy7 (B159), anti-CD25-PE (M-251) purchased from BD Pharmigen; anti-CD16-PE (3G8), anti-MICA/B-BV650 (6D4), anti-ULBP2/5/6-PE (165903), anti-CD155/PVR-BV605 (SKII.4), anti-CD112/Nectin-2-PE (R2.525), anti-CD95/FAS-BV421 (DX2), anti-CD262/TRAIL/R2-PE (YM366), anti-NKG2A-FITC (REA110), anti-NKG2C-BV510 (134591), anti-TRAIL-BV421 (RIK-2), anti-CD314/NKG2D-BV605 (1D11) purchased from BD Biosciences; anti-ULBP1-PE (170818), anti-ULBP3-PE (166510), anti-CD155/PVR-PE (300907), anti-CD112/Nectin-2-APC (610603), anti-TRAIL/R2-APC (17908), anti-CD226/DNAM-1-PE (102511), anti-KIR3DL1-APC (DX9), purchased from R&D System; anti-CD337/NKp30-PerCP-Cy5.5 (P30-15), anti-CD335/NKp46-BV421 (9E2) purchased from Sony; anti-CD152/CTLA-4-APC (14D3), anti-TIGIT-APC (MBSA43), anti-PD-1-APC (eBioJ105), anti-FAS (SM1/23) purchased from Invitrogen; anti-MHC-I-FITC (W6/32) purchased from RayBiotech; anti-CD14-AlexaFluor700 (HCD14), anti-CD19-AlexaFluor700 (HIB19), anti-CD54/ICAM-1-BV421 (HA58) purchased from BioLegend; anti-CD45-PE-Cy5 (MEM-28) purchased from ExBio; anti-FAS-L-APC (NOK-1) purchased from Abcam; anti-KIR2DL1/2DS1-PC-5.5 (EB6B), anti-KIR2DL2/L3/S2-PE (GL-183), purchased from Beckman Coulter; anti-CD57-FITC (G683), purchased from Abbexa. Neutralizing experiments were performed with the following antibodies: anti-FAS-L (NOK-1) purchased from Biolegend, anti-TRAIL/TNFSF10, anti-ICAM-1 (11C81) purchased from R&D System; anti-LFA-1 (TS-1) purchased from Biocompare.

Flow cytometry data were acquired using Cytoflex (Beckman Coulter) and analyzed by FlowJo Software, version 10.0.8r1 (Treestar, Ashland, OR, USA), or CytExpert version 2.5 software.

### BC cell lines, spheroid formation and cytokine treatment

The human BC cell lines MCF-7, MDA-MB-231, and MDA-MB-468 were provided by ATCC and used in this study. All BC cell lines were characterized by HLA class I typing by PCR-SSP sets (Genovision As, Oslo, Norway) according to the instructions of the manufacturer. Cell lines were routinely checked for *Mycoplasma* contamination prior to the use. MCF-7 cells are p53 WT and are luminal-A, while MDA-MB-231 and MDA-MB-468 cells are p53 mutant and TNBC. The BC cell lines were cultured in complete DMEM medium supplemented with 10% FBS (Thermo Fisher Scientific, Waltham, MA, USA), 2 mM glutamine, 100 mg/mL penicillin and 50 mg/mL streptomycin (Euro Clone S.p.a., Milan, Italy). BC cell lines cultured in 2D were treated with a cytokine cocktail containing 50 ng/mL IFN-γ (285-IF, R&D) and 20 ng/mL TNF-α (210-TA, R&D) for 48 h.

For spheroid formation, BC cells were seeded in Nunclon Sphera 96-well U-bottom ultra-low attachment plates (Thermo Fisher) at a density of 1 × 10⁴ cells per well. After 24 h, spheroids were formed, as confirmed by optic microscopy. Since the MDA-MB-468 cell line, unlike MCF-7 and MDA-MB-231, does not form spheroids, it was used in the 2D experiments only.

BC spheroids were either untreated or treated with a low-dose, non-toxic cytokine cocktail, with cytokine concentration previously established by apoptotic dose-response assays (75 ng/mL IFN-γ and 30 ng/mL TNF-α) for 24 h and added of medium as control or 25 × 10^3^ NK cells per spheroid for another 24 h. After 24 h of co-culture with NK cells, spheroids were visualized under a light microscopy (Olympus IX microscope) at a magnification of 10X and their size was measured using the Capture 3.0 software. For functional assays, after 24 h of co-culture, five to eight wells of NK cell-BC spheroid co-cultures were collected for each condition and gently washed with a cell strainer to separate non-infiltrating NK cells (passed through the cell strainer) and spheroids containing infiltrated NK cells (retained by cell strainer). Subsequently, non-infiltrating NK cells and the spheroids, mechanically disaggregated, were stained with a cocktail of antibodies to evaluate, in different experiments, (i) NK cell percentage, (ii) expression of receptors in non-infiltrated and infiltrated NK cells, (iii) the degranulation of non-infiltrated and infiltrated NK cells, (iv) the apoptotic state of BC spheroid cells.

### Human NK cells, Apoptosis and Degranulation assay

Human NK cells were isolated from peripheral blood of healthy donors, in accordance with the Institutional Review Board of Bambino Gesù Children’s Hospital, IRCCS, Rome-Italy, by RosetteSep NK-cell enrichment mixture method Kit (StemCell Technologies, Vancouver, BC, Canada) and Ficoll-Paque Plus (Lympholyte Cedarlane, Burlington, ON, Canada) centrifugation. NK cells were routinely checked for CD3^-^ CD14^-^ CD19^-^ CD56^+^ immunophenotype and the expression of a panel of inhibitory/activating KIRs such as KIR2DL1/S1, KIR2DL2/L3/S2, and KIR3DL1, activating receptors such as DNAM-1, NKG2D, NKp30, NKp46 and CD25, differentiation marker such as CD57 and NKG2C, inhibitory receptor NKG2A, immune checkpoint receptors such as TIGIT, PD-1 and CTLA-4, and dead molecules such as FAS-L and TRAIL was evaluated by flow cytometry. The gate strategy adopted to analyze NK cells is shown in Supplementary Fig. S1. NK cells with greater than 90% purity were resuspended in NK MACS medium (Miltenyi Biotec, Bergisch Gladbach, Germany), supplemented with NK MACS supplement, AB serum, and 500 IU/mL of recombinant human IL-2 (PeproTech, Rochy Hill, NJ, USA) and cultured at 37 °C, divided every three days, and used for up to 20 days after isolation.

The K562 cell line (ATCC), used as a control target in NK cell degranulation assays, was cultured in RPMI 1640 medium supplemented with 10% FBS (Thermo Fisher Scientific, Waltham, MA, USA), 2 mM glutamine, 100 mg/mL penicillin, and 50 mg/mL streptomycin (Euro Clone S.p.A., Milan, Italy).

The apoptotic state of tumor cells was evaluated in BC cell lines co-cultured with NK cells in a 1:1 effector (E):target (T) ratio for 4 h at 37 °C and 5% CO_2_. After the co-culture, cells were stained with anti-CD56-PE-Cy7 for 20 min, washed, and resuspended in apoptosis detection kit buffer (Biolegend, San Diego, CA, USA) containing PE-conjugated Annexin V and 7-AAD. The apoptotic state of BC cells was evaluated in CD56^-^ cell subset by flow cytometry analysis. For some apoptotic assays on 2D tumor cell cultures, BC cell lines, untreated or pretreated with IFN-γ and TNF-α (as described above), were incubated with 100 μM pan-caspase Z-VAD-FMK inhibitor (Selleck Chemicals) for one hour before being co-cultured with NK cells. For blocking experiments, NK cells were pretreated for one hour with 10 μg/ml of neutralizing anti-FAS-L, or 1 μg/ml of neutralizing anti-TRAIL, or 20 μg/ml of neutralizing anti-LFA-1, or a cocktail of all three antibodies before to be co-cultured with MDA-MB-231 cells.

To assess the apoptotic state of BC spheroid cells, after 24 h of co-culture with NK cells and separation by cell strainer washing (as described above), disaggregated BC spheroids were stained with anti-CD56-PE-Cy7 for 20 min, washed, and resuspended in apoptosis detection kit buffer containing PE-conjugated Annexin V and 7-AAD, and evaluated in CD56^-^ cell subset by flow cytometry analysis.

To perform the degranulation assays, NK cells were co-cultured with BC cells at 1:1 E:T ratio for 3 h in complete medium in the presence of anti-CD107a at a 1:100 dilution. During the last 2 h of co-culture, GolgiStop (BD Bioscence), used at a 1:500 dilution, was added. Cells were then washed, centrifuged, and stained with anti-CD56, anti-CD16, anti-CD3 and anti-CD45 to evaluate CD107a expression in the CD56^+^CD16^+^CD45^+^CD3^-^ subset by flow cytometry [15]. To assess the degranulation of NK cells non-infiltrated and infiltrated in BC spheroids, NK cells were added to spheroids, as described above, in presence of anti-CD107a at a 1:100 dilution. After one hour of co-culture, GolgiStop, used at a 1:500 dilution, was added. After 18 h of co-culture, non-infiltrating NK cells and infiltrating NK cells, separated as described above, were stained with a cocktail of antibodies to evaluate CD107a expression in the CD56^+^CD16^+^CD45^+^CD3^-^ subset by flow cytometry.

### Conjugation assay

For conjugate formation assay, NK cells were stained with PKH26 red fluorescent dye (at a 1:1000 dilution, Invitrogen), while BC cells, untreated or pretreated with IFN-γ + TNF-α, with CFSE fluorescent dye (at a 1:2000 dilution, Invitrogen) for 30 min at 37 °C. Then, cells were washed by adding FBS and PBS to interrupt the staining. To promote conjugate formation, PKH26-stained NK cells and CFSE-stained BC cells were suspended at 2 × 10^5^ cells each cell type in 100 μl of complete DMEM medium, and co-cultured in 1.5 ml eppendorf, at 1:1 ratio for one hour at 37 °C. The reaction was stopped by fixing cells with 1% paraformaldehyde (PFA, Sigma-Aldrich) and conjugate formation was evaluated by flow cytometry. For blocking experiments, CFSE-stained MDA-MB-231 cells were pretreated for one hour with 20 μg/ml of neutralizing anti-ICAM-1, while PKH26-stained NK cells were pretreated with 20 μg/ml neutralizing anti-LFA-1, or with medium as control, before being co-cultured together for one hour at 37 °C for conjugate formation evaluated as described above.

### Caspase 3/7 assay

In addition to the Annexin V/7AAD kit, NK cell-mediated apoptosis of BC spheroids was evaluated by caspase-3/7 Glo assay (Promega Italia, Milano, Italy), a luminescent assay that measures caspase-3 and -7 activities present in apoptotic cells. MDA-MB-231 and MCF-7 spheroids, untreated or treated with IFN-γ + TNF-α, were co-cultured with NK cells, as described above, in 100 µl of complete DMEM medium. Then, the Caspase-Glo® 3/7 Reagent was added to spheroid/NK cell co-cultures at 1:1 ratio in a final volume of 200 µl. Plates were incubated at 37 °C for 30 min and the luminescence of each sample was detected by the plate-reading luminometer Glomax reader (Promega Italia, Milano, Italy) and analyzed by ProNect™ Application Files.

### Acquisition of fluorescence images of spheroids

MDA-MB-231 spheroids were generated as described above and untreated or treated with IFNγ + TNFα for 24 h. Then, spheroids were co-cultured with NK cells, pre-stained with CFSE (at a 1:1000 dilution, for 30 min at 37 °C), for 16 h, and subsequently fixed in 4% PFA. Following, spheroids were incubated with penetration buffer containing 0.1% Triton X-100 in PBS 1X for 16 h, washed in PBS 1× and stained with DAPI (D3571; 1:1000; Invitrogen) for 30 min. Spheroids were then resuspended in mounting medium (Ibidi, Cat. 50001) and transferred into µ-Slide 8 well glass bottom (Ibidi, Cat. 80807).

The Axio Observer Z1 inverted microscope, equipped with an ApoTome.2 System (Carl Zeiss Inc., Ober Kochen, Germany) was used for the spheroid images acquisition. Optical slices were obtained at adequate intervals on the Z-axis (between 50 µm and 500 µm). Slice and orthogonal projections were obtained using ZEN 3.8 Pro software (Zeiss). Maximum z-projections were made using ImageJ (Fiji) software (https://imagej.net/software/fiji/).

### Statistical Analysis

Statistical analysis was performed by GraphPad software. Data were analyzed using the unpaired two-tailed Student’s *t*-test. A *p*-value of <0.05 was considered statistically significant.

## Results

### Effect of IFN-γ + TNF-α-treatment on the expression of ligands for NKARs, death receptors and ICAM-1 in BC cells

**Fig. 1:**
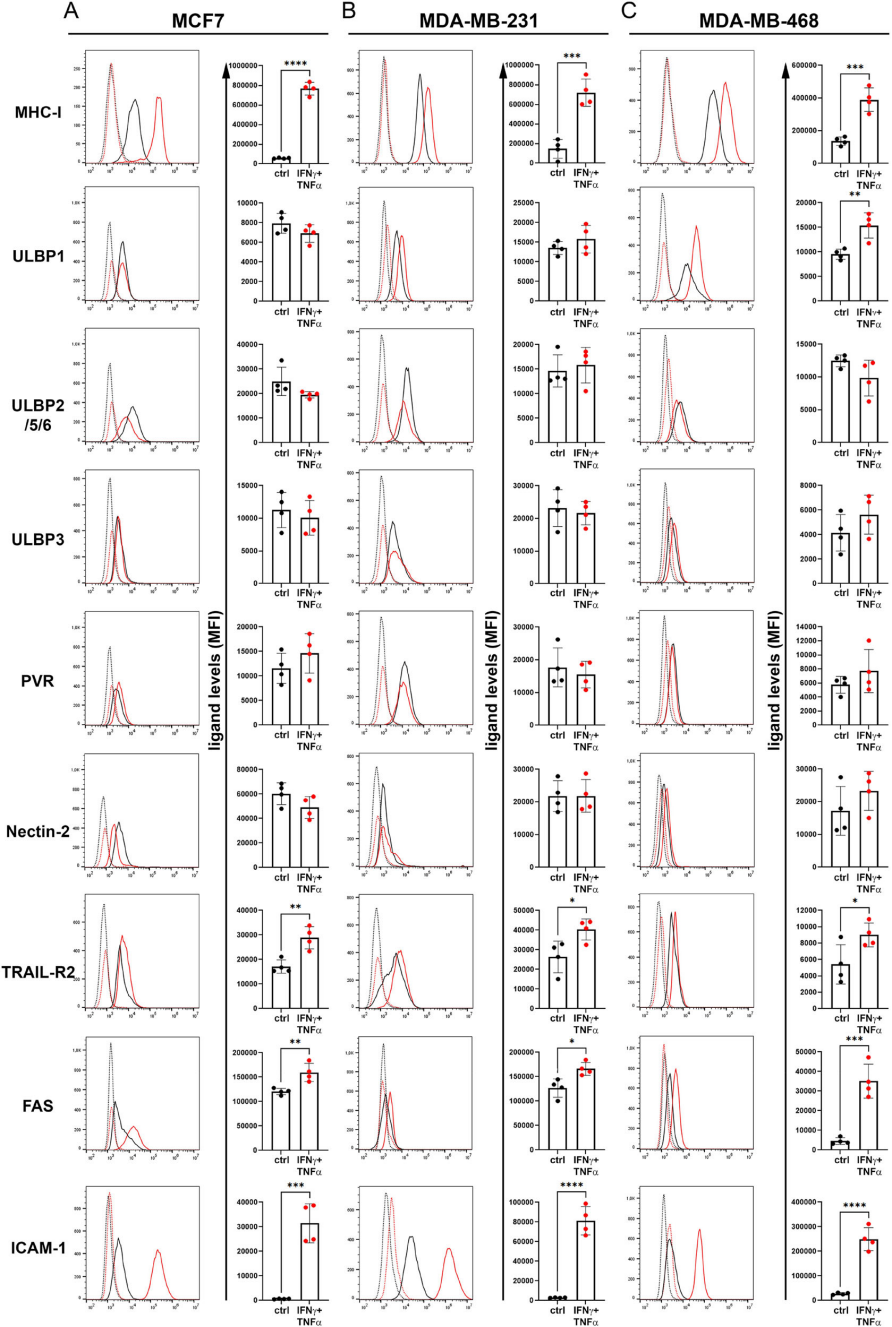
Effect of IFN-γ + TNF-α-treatment on the expression of ligands for NK cell-activating receptors, death receptors TRAIL-R2 and FAS, and the adhesion molecule ICAM-1 in BC cell lines. MCF-7 (**A**), MDA-MB-231 (**B**), and MDA-MB-468 (**C**) BC cell lines were untreated or treated for 24 h with non-toxic low doses of IFN-γ + TNF-α and then evaluated for surface expression of MHC class I and the indicated molecules by flow cytometry. Each panel shows a representative (left) and a summary of four independent experiments (right). Black or red lines (dashed for isotype control antibodies, solid for specific antibodies) are displayed for untreated or IFN-γ + TNF-α-treated BC cell lines, respectively (left). Normalized mean of fluorescence (MFI) for each experiment was displayed by comparing the IFN-γ + TNF-α-treated BC cell lines (red dots) with the untreated or control (black dots) cell lines (right). Mean ± SD; *p < 0.05, **p < 0.01,***p < 0.001; *p* value, two tailed unpaired Student-*t* test.

As a first approach, we addressed whether treatment with non-toxic, low doses of IFN-γ + TNF-α could have immunomodulatory effects on BC cells and thus modulate their susceptibility to NK cell-mediated recognition and killing. MCF-7, MDA-MB-231 and MDA-MB-468 cell lines were treated with IFN-γ + TNF-α for 48 h and the expression of ligands for the NKG2D (ULBP1, ULBP2/5/6 and ULBP3) and DNAM-1 (PVR and Nectin-2), as well as two death receptors (TRAIL-R2 and FAS) and the adhesion molecule ICAM-1, was evaluated by flow cytometry (Fig. 1). The efficacy of cytokine treatment was monitored by evaluating the expression of the major histocompatibility complex (MHC) class I (MHC-I), which is known to be overexpressed in many tumor cell lines upon treatment with these cytokines [15, 22]. Treatment with IFN-γ + TNF-α significantly increased ULBP1 expression only in MDA-MB-468, while all other ligands remained unchanged in all three BC cell lines (Fig. 1). Consistent with this evidence, the degranulation of NK cells cocultured with cytokine-treated BC cell lines remained unchanged as compared to that of NK cells co-cultured with untreated BC cells (Supplementary Fig S2), thus confirming that IFN-γ + TNF-α-treatment did not affect the recognition of BC cells by NK cells.

**Fig. 2:**
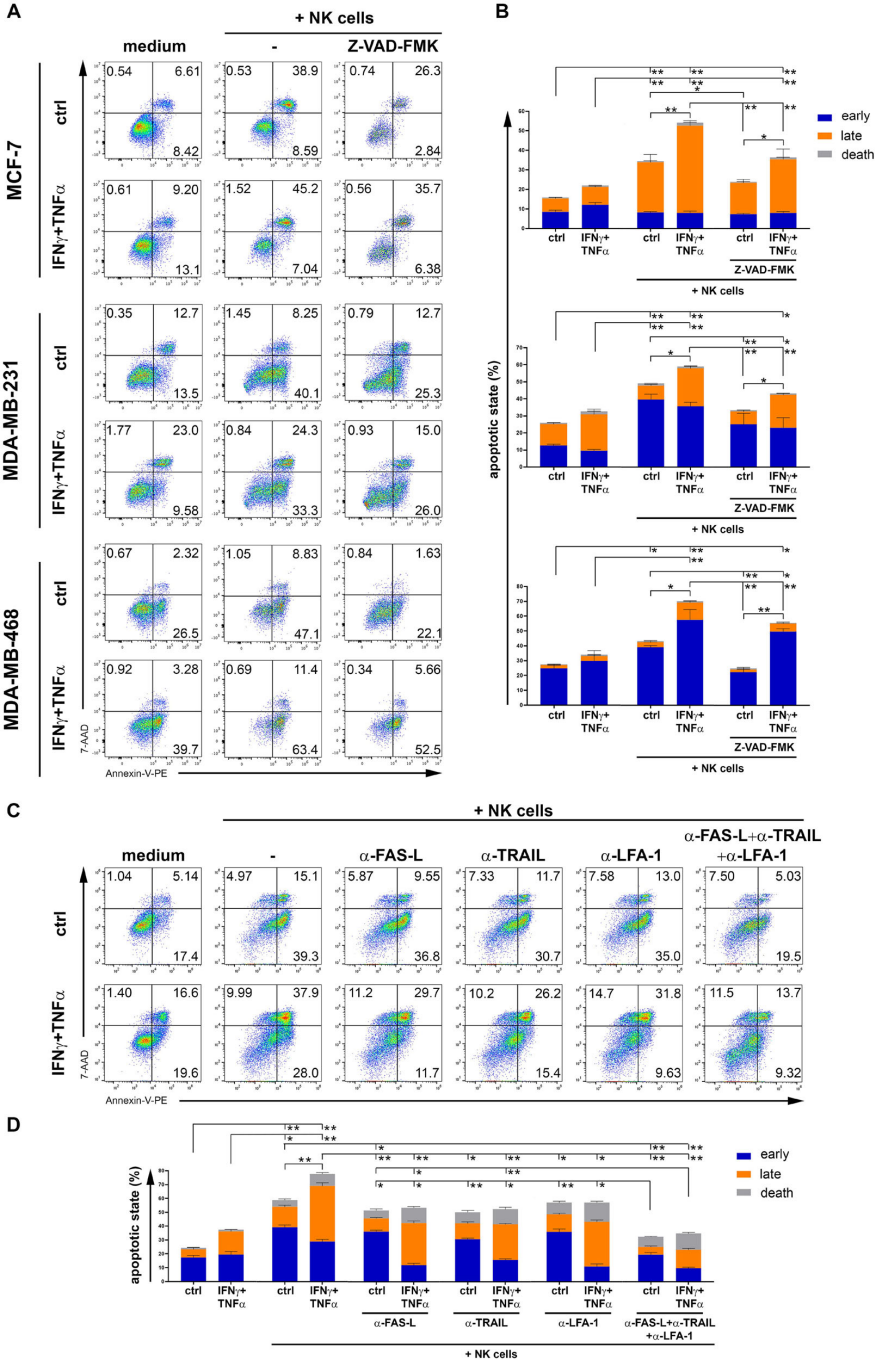
Enhancement of NK cell-mediated apoptosis of BC cell lines following treatment with IFN-γ + TNF-α. **A** MCF-7, MDA-MB-231 and MDA-MB-468 BC cell lines were untreated or treated for 24 h with low, non-toxic doses of IFN-γ + TNF-α and then used as target cells for NK cell-apoptotic assay. Untreated or IFN-γ + TNF-α-treated BC cell lines without NK cells were used as negative controls, while untreated or IFN-γ + TNF-α-treated BC cell lines co-cultured with Z-VAD-FMK pre-treated NK cells were used as apoptosis controls. A representative experiment of the four performed (**B**) is shown for each indicated BC cell line. The percentage of cells in early apoptosis (Annexin V^+^ 7-AAD^-^), late apoptosis (Annexin V^+^ 7-AAD^+^) and dead state (Annexin V^-^ 7-AAD^+^) are shown for each plot. **B** A summary of four independent experiments is shown. **C** MDA-MB-231 BC cell lines were untreated or treated for 24 h with low, non-toxic doses of IFN-γ + TNF-α and then used as target cells for neutralizing NK cell-apoptotic assay by pre-incubating NK cells with neutralizing antibodies such as α-FAS-L, α-TRAIL, α-LFA-1 or a cocktail of all three antibodies, before co-culturing them with BC cells. A representative experiment of the four performed with MDA-MB-231 cell line (**D**) is shown. The percentage of cells in early apoptosis, late apoptosis and dead state are shown for each plot. **D** A summary of four independent experiments is shown. Mean ± SD; *p < 0.05, **p < 0.01; *p* value, two tailed unpaired Student-*t* test.

In contrast, treatment with IFN-γ + TNF-α induced a significant increase in the expression of TRAIL-R2, FAS and ICAM-1 in all three BC cell lines (Fig. 1). To assess whether the increased expression of these molecules could be reflected in the function of NK cells, we performed a co-culture of cytokine-treated BC cell lines with NK cells and assessed the apoptotic state of BC cells by Annexin-V and 7-AAD staining (Fig. 2A, B). In all three BC cell lines the treatment with IFN-γ + TNF-α significantly increased NK cell-mediated apoptosis. This effect was reduced by the pan-caspase inhibitor Z-VAD-FMK, consistent with an increased activation of the apoptotic pathway triggered by the up-modulation of TRAIL-R2 and FAS on cytokine-treated BC cells (Fig. 1). To evaluate the role of TRAIL-R2, FAS and ICAM-1 in increasing NK cell-mediated apoptosis of cytokine-treated BC cell lines, we performed neutralization assay by pre-incubating NK cells with antibodies that recognize TRAIL, FAS-L and anti-LFA-1 which are ligands for TRAIL-R2, FAS and ICAM-1 respectively (Fig. 2C, D) [23–25]. NK cell-mediated apoptosis of cytokine-treated MDA-MB-231 was significantly reduced when NK cells were pre-treated with single anti-FAS-L, anti-TRAIL and anti-LFA-1 antibodies, and completely abolished with a cocktail of the three antibodies, thus indicating that all three FAS, TRAIL-R2 and ICAM-1 are synergistically involved in NK cell-mediated apoptosis of cytokine-treated BC cell lines.

**Fig. 3:**
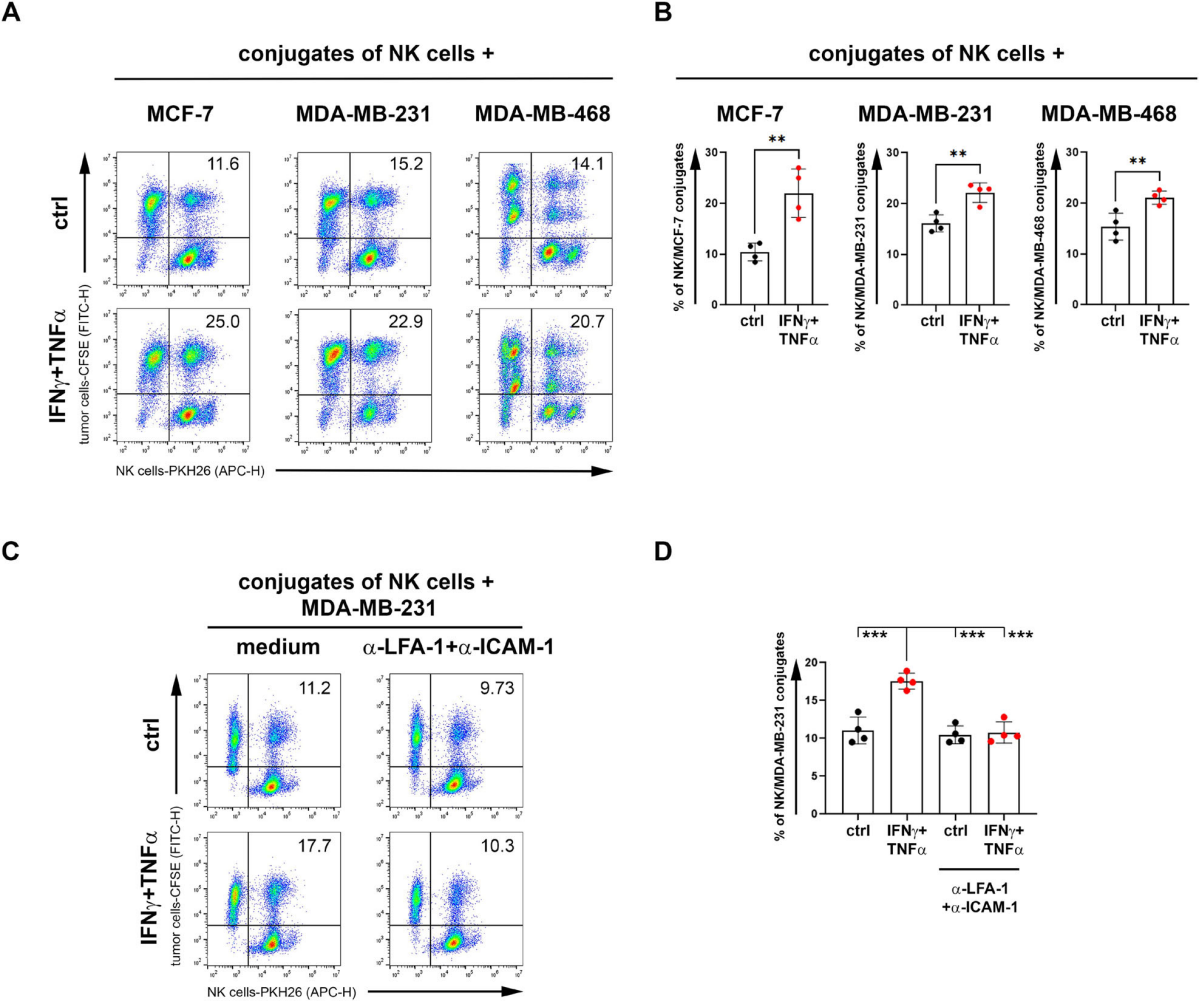
Increased percentage of conjugates between NK cells and BC cell lines following treatment with IFN-γ + TNF-α. **A** BC cell lines were treated with IFN-γ + TNF-α as described above, stained with CFSE and co-cultured with PKH26-stained NK cells for one hour. The percentage of CFSE^+^PKH26^+^ cells representing BC-NK cell conjugates was reported in each plot. **B** The summary of four independent experiments was reported. **C** The neutralizing conjugation assay was performed by incubating CFSE-stained MDA-MB-231 with neutralizing α-ICAM-1 and PKH26-stained NK cells with neutralizing α-LFA-1 for one hour before culturing them together as described above. The percentage of BC-NK cell conjugates was reported in each plot. **D** The summary of four independent experiments was reported. Mean ± SD; **p < 0.01, ***p < 0.001; *p* value, two tailed unpaired Student-*t* test.

Furthermore, to functionally assess the increase in ICAM-1 expression on BC cells induced by IFN-γ + TNF-α-treatment (Fig. 1), we performed conjugate formation assays by co-culturing NK cells with cytokine-treated BC cell lines. Indeed, the percentage of conjugates formed by NK cells with all three cytokine-treated BC cell lines was significantly increased (Fig. 3A, B and Supplementary Fig. S3). To assess if the ICAM-1 up-modulation in cytokine-treated BC cell lines could affect the conjugate formation with NK cells, we performed neutralizing assay by using both anti-ICAM-1 and anti-LFA-1 (Fig. 3C, D). As shown in Fig. 3C, D, neutralizing ICAM-1 on MDA-MB-231 cells and LFA-1 on NK cells, the percentage of conjugates formed by NK cells with cytokine-treated MDA-MB-231 cells was significantly reduced to the level of the control ones. These results indicate that IFN-γ + TNF-α-treatment of BC cell lines improved the binding between NK cells and BC cells through the modulation of the adhesion molecule ICAM-1, thus supporting NK cell-mediated activity against BC cells.

### NK cell infiltration-ability in BC spheroids treated with IFN-γ + TNF-α

**Fig. 4:**
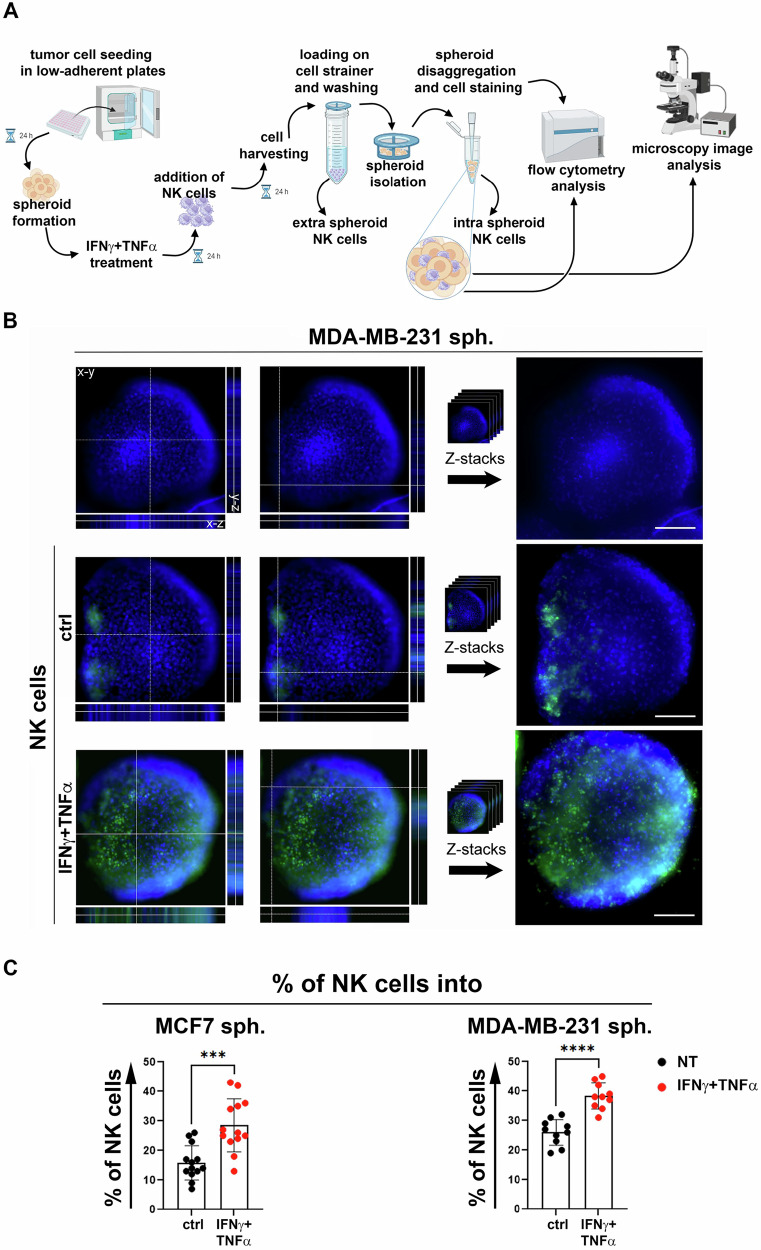
Treatment with IFN-γ + TNF-α enhanced NK cell infiltration into BC spheroids. **A** MCF-7, MDA-MB-231 and MDA-MB-468 BC cell lines were seeded in low-adherent plates for 24 h to obtain spheroids. The MDA-MB-468 cell line did not produce spheroids (Supplementary Figure 4). Then, MCF-7, MDA-MB-231 spheroids were either untreated or treated for 24 h with low, non-toxic doses of IFN-γ + TNF-α and supplemented with NK cells for an additional 24 h. Then, the cell/spheroid suspensions were loaded on a cell strainer and washed with medium to isolate the spheroids from non-infiltrated (extra spheroids) NK cells. The spheroids were disaggregated to obtain single cell suspensions and were stained with a cocktail of antibodies to evaluate the NK cells infiltrated in the spheroids by flow cytometry. In some experiments spheroid images were acquired by microscopic analysis to assess spheroid size and NK cell infiltration. **B** Orthogonal view of NK cell infiltration into representative MDA-MB-231 spheroids. Orthogonal view (left) and maximum z-projection (Z-stacks, right) of spheroid alone or co-cultured with NK cells, untread (ctrl) or treated with IFNγ + TNFα. NK cells were stained with CFSE (green). DAPI was used for nuclear staining (blue). Bars correspond to 100 µm. **C** The percentage of CD45^+^CD56^+^CD16^+^CD3^-^ NK cells infiltrated in untreated BC spheroids (ctrl, black dots) or IFN-γ + TNF-α-treated (IFN-γ + TNF-α, red dots) was evaluated. A summary of thirteen and ten independent experiments on MCF-7 and MDA-MB-231 spheroids, respectively, is reported. MFI, mean of fluorescence intensity. Mean ± SD; ***p < 0.001, ****p < 0.0001; *p* value, two tailed unpaired Student-*t* test.

Next, to assess the effect of IFN-γ + TNF-α in a 3D tumor model such as BC spheroids and the effect of the cytokines on the susceptibility of spheroids to NK cell-mediated killing, MCF-7, MDA-MB-231 and MDA-MB-468 cells were cultured in low-adherent plates. However, we obtained spheroids only from MCF-7 and MDA-MB-231, but not from MDA-MB-468 cells (Supplementary Fig. S4). MCF-7 and MDA-MB-231 spheroids were treated with low, non-toxic doses of IFN-γ + TNF-α for 24 h and co-cultured with NK cells for another 24 h. Then, BC spheroids were separated from the non-infiltrating NK cells (extra-spheroid NK cells) by passing and washing the cell/spheroid suspension through a cell strainer. Finally, the infiltration of NK cells (intra-spheroid NK cells) into BC spheroids was assessed by disaggregating the spheroids, staining the disaggregated cells with a cocktail of antibodies for NK cell receptors and analyzing them by flow cytometry. Alternatively, spheroids images were acquired by fluorescence microscopy (Fig. 4A). Fluorescence microscopy z-stack analysis revealed a clear increase in the presence of NK cells in IFN-γ + TNF-α-treated spheroids compared with untreated spheroids (Fig. 4B and Supplementary Fig S5). Furthermore, the percentage of infiltrated NK cells increased significantly in MCF-7 and MDA-MB-231 spheroids treated with IFN-γ + TNF-α compared to untreated spheroids (Fig. 4C). These results indicate that treatment with IFN-γ + TNF-α rendered BC spheroids significantly more accessible to NK cells, thus suggesting that an immune mechanism was triggered to overcome the resistance that typically occurs in the 3D tumor structure [11, 26].

### Immunophenotype of NK cells infiltrated and non-infiltrated in BC spheroids treated with IFN-γ + TNF-α

**Fig. 5:**
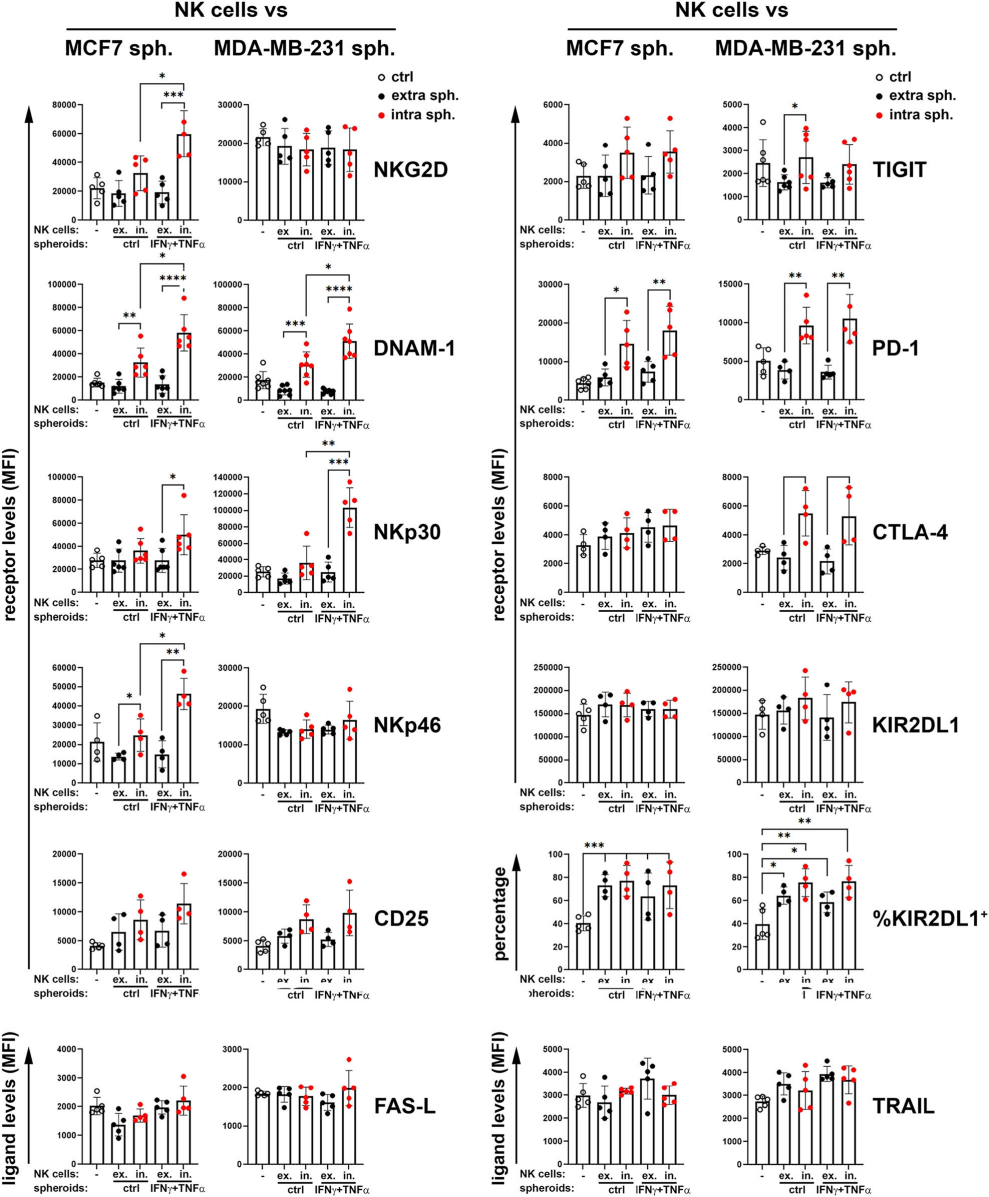
Expression of receptors on NK cells non-infiltrated or infiltrated into untreated or IFN-γ + TNF-α-treated BC spheroids. After co-culturing NK cells with BC spheroids, extra spheroids NK cells were separated from intra-spheroids NK cells (as described above) and stained for the expression of NKARs, NCRs, CD25, TIGIT, immune check-point molecules, KIRs and death receptors. The expression of the indicated NK cell surface molecules was evaluated in CD45^+^CD56^+^CD16^+^CD3^-^ NK cells alone (empty dots) as controls, not infiltrated (extra sph., ex., black dots) or infiltrated (intra sph., in., red dots) into untreated (ctrl) or IFN-γ + TNF-α-treated (IFN-γ + TNF-α) BC spheroids. The summary of four independent experiments was reported for each molecule. MFI, mean of fluorescence intensity. Mean ± SD; *p < 0.05, **p < 0.01; ***p < 0.001, ****p < 0.0001; *p* value, two tailed unpaired Student-*t* test.

The immunophenotype of NK cells, infiltrated (intra, in.) or non-infiltrated (extra, ex.) in both MCF-7 and MDA-MB-231 spheroids was assessed by measuring the expression levels of (i) NKARs such as NKG2D and DNAM-1; (ii) natural cytotoxic receptors (NCRs) such as NKp30 and NKp46; (iii) the interleukin-2 receptor CD25; (iv) the inhibitory receptor TIGIT; (v) the inhibitory check-point molecules PD-1 and CTLA-4; (vi) the inhibitory receptor KIR2DL1; and finally the ligands for death receptors such as FAS-L and TRAIL (Fig. 5).

NKG2D expression was significantly increased in NK cells infiltrated in MCF-7 spheroids treated with IFN-γ + TNF-α compared to untreated spheroids. Under the same conditions, a significant increase in DNAM-1 expression was found in NK cells infiltrated in MCF-7 and MDA-MB-231 spheroids treated with IFN-γ + TNF-α compared to untreated spheroids. It was also assessed that, regardless of cytokine treatment, NKG2D levels on NK cells infiltrating MCF-7 spheroids and DNAM-1 levels on NK cells infiltrating both MCF-7 and MDA-MB-231 spheroids were significantly higher than those on NK cells non-infiltrating spheroids (Fig. 5A). This finding suggests a higher activation state of NK cells infiltrating BC spheroids versus those remaining outside the spheroids.

NKp30 expression was significantly increased in NK cells infiltrated in MDA-MB- 231 spheroids treated with IFN-γ + TNF-α compared to untreated spheroids. Under the same conditions, a similar trend was found in NK cells infiltrated in MCF-7 spheroids. The increase in NKp30 was also significant in NK cells infiltrated in MCF-7 and MDA-MB-231 spheroids treated with IFN-γ + TNF-α versus non-infiltrated NK cells, thus corroborating the superior activation immunophenotype of spheroid-infiltrating as compared to non-infiltrating NK cells (Fig. 5A). In addition, NKp46 expression was significantly increased on NK cells infiltrated in MCF-7 spheroids treated with IFN-γ + TNF-α compared to untreated spheroids. Moreover, regardless of treatment, a significant increase in NKp46 was assessed on spheroid-infiltrating compared to non-infiltrating NK cells, further supporting the superior activation state of intra-spheroid compared to extra-spheroid NK cells. In contrast, no significant differences in NKp46 expression were observed on NK cells co-cultured with control or cytokine-treated MDA-MB-231 spheroids (Fig. 5A). These data indicate that the expression of NKp46 and NKG2D receptors on NK cells may be conditioned differently depending on the type of BC cells, i.e. TNBC (MDA-MB-231) versus luminal-A (MCF-7) cells, with which they come in contact, in agreement with the greater resistance to different treatments typical of TNBCs as compared to luminal-A cells [2, 3]. The expression of CD25 on spheroid-infiltrating NK cells showed a non-significant increase compared to non-infiltrating NK cells, regardless of IFN-γ + TNF-α-treatment (Fig. 5A).

Furthermore, irrespective of cytokine treatment, the expression of TIGIT on NK cells infiltrated in MCF-7 spheroids showed a non-significant increase compared to non-infiltrated ones. Similarly, TIGIT expression increased on NK cells infiltrated in MDA-MB-231 spheroids compared to non-infiltrated ones, but the increase was significant only in those co-cultured with untreated spheroids. This finding indicated that spheroid-infiltrating NK cells, not only showed an immunophenotype indicative of increased stimulation, but also displayed a phenotype coherent with an increased state of exhaustion, typical of NK cells within 3D tumor structure and in the TME [27, 28] (Fig. 5B).

Additionally, the expression of PD-1 and CTLA-4 and KIR2DL1 was assessed. PD-1 expression was significantly increased in infiltrated versus non-infiltrated NK cells, regardless of cytokine treatment, in both MCF-7 and MDA-MB-231 spheroids. A similar pattern was obtained for CTLA-4 expression in infiltrated versus non-infiltrated NK cells in MDA-MB-231 but not in MCF-7 spheroids. According to these data, spheroid-infiltrating NK cells showed an immunophenotype characterized by a state of activation and exhaustion with high expression levels of NKARs and inhibitory check-point molecules; moreover, similarly to NKp46 and NKG2D, (Fig. 5A), CTLA-4 expression on NK cells was differently influenced by the BC cell type (Fig. 5B). This finding confirms the greater immune escape ability of TNBCs (MDA-MB-231) compared to luminal-A (MCF-7) cells.

Regarding the expression of KIR2DL1, no significant changes were found in infiltrated versus non-infiltrated KIR2DL1^+^ NK cells in both types of BC spheroids. However, when analyzing the percentage of KIR2DL1^+^ versus KIR2DL1^-^ NK cells, regardless of cytokine treatment, a non-significant increase of KIR2DL1^+^ NK cells emerged between spheroid-infiltrating versus non-infiltrating NK cells in both MCF-7 and MDA-MB-231 spheroids. Furthermore, the percentages of KIR2DL1^+^ NK cells, infiltrated and non-infiltrated in MCF-7 and MDA-MB-231 spheroids, were significantly higher than control NK cells not in contact with spheroids (Fig. 5B). This finding indicates that spheroids-infiltrating NK cells exhibited an immunophenotype suggestive of an activated state typical of KIR-expressing NK cells [29, 30], simultaneously to a state of exhaustion as described above.

Finally, the expression of FAS-L and TRAIL on spheroid-infiltrating NK cells remained unchanged compared to non-infiltrating NK cells, regardless of IFN-γ + TNF-α-treatment (Fig. 5A, B).

Overall, these results show that treatment of BC spheroids with IFN-γ + TNF-α resulted in an increased expression of receptors NKG2D, DNAM-1, NKp30 and NKp46 in spheroid-infiltrating NK cells, suggesting the induction of an enhanced state of activation. However, the increase of PD-1 and CTLA-4, regardless of treatment, indicated that the enhanced activation of spheroids-infiltrating NK cells was accompanied by increased exhaustion. The immunophenotype of NK cells varied based on the receptors, reflecting changes in their activation and function depending on tumor type (TNBCs versus luminal-A) with which they were in contact, and their ability to infiltrate 3D structures. These data therefore highlight the importance of the TME in modulating the response of NK cells and their ability to recognize and kill tumor cells within 3D structures.

### Immunomodulatory effects of IFN-γ + TNF-α-treatment on BC spheroids

**Fig. 6:**
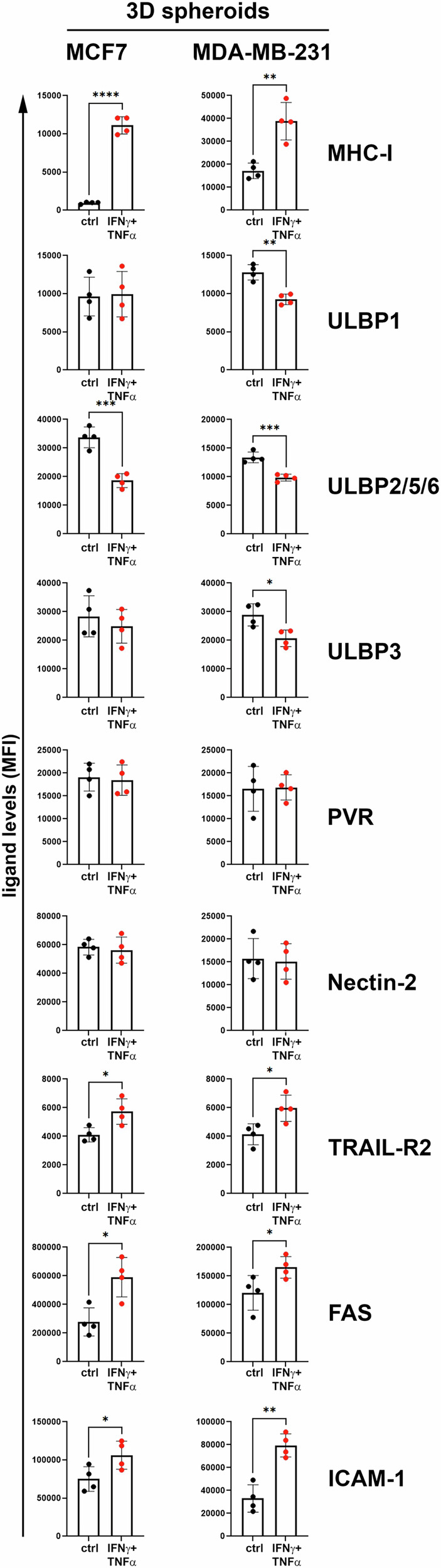
Effect of IFN-γ + TNF-α on the expression of ligands for NK cell-activating receptors, death receptors FAS and TRAIL-R2, and the adhesion molecule such as ICAM-1 in BC spheroids. MCF-7 and MDA-MB-231 BC spheroids were either untreated (ctrl, black dots) or treated for 48 h with low non-toxic doses of IFN-γ + TNF-α (IFN-γ + TNF-α, red dots), disaggregated and then stained to evaluate surface expression of the indicated molecules by flow cytometry. A summary of four independent experiments is reported for each molecule. Normalized mean of fluorescence (MFI) values for each experiment were displayed. Mean ± SD; *p < 0.05, **p < 0.01; ***p < 0.001, ****p < 0.0001; *p* value, two tailed unpaired Student-*t* test.

Next, we investigated the effect of IFN-γ + TNF-α-treatment on the expression of ligands for the NKG2D and DNAM-1 NKARs as well as on the expression of FAS, TRAIL-R2 and ICAM-1 in MCF-7 and MDA-MB-231 spheroids (Fig. 6). As assessed for the BC cell lines in 2D cultures (Fig. 1), the expression of the MHC-I molecule was evaluated as a positive control for IFN-γ + TNF-α-treatment. ULBP1 expression showed no change in cytokine-treated MCF-7 spheroids, while it was significantly reduced in MDA-MB-231 spheroids. Differently, ULBP2/5/6 expression showed a significant decrease in MCF-7 and MDA-MB-231 spheroids following cytokine treatment. ULBP3 expression showed no change in cytokine-treated MCF-7 spheroids, while it was significantly reduced in MDA-MB-231 spheroids treated with IFN-γ + TNF-α compared to controls.

The ligands recognized by DNAM-1, such as PVR and Nectin-2, showed no significant changes in expression between control and IFN-γ + TNF-α-treated spheroids in both BC cell lines.

In contrast to ligands recognized by NKARs, under IFN-γ + TNF-α-treatment conditions, a significant increase in the expression of TRAIL-R2, FAS, and ICAM-1 was detected not only in MCF-7 but also in MDA-MB-231 spheroids, thus underling, as reported above for 2D cultures (Fig. 1), the cytokine immunomodulatory effects on MDA-MB-231, a cell line model of aggressive TNBC [31].

Furthermore, both MCF-7 and MDA-MB-231 spheroids treated with IFN-γ + TNF-α showed a significant increase in ICAM-1 expression, similar to the findings obtained in 2D BC cell cultures (Fig. 1). These results indicate that the induced increase in ICAM-1 mediated by IFN-γ + TNF-α-treatment not only facilitated NK cell-BC cell contact and conjugate formation (Fig. 3) but also promoted the increased infiltration of NK cells into BC spheroids (Fig. 4B, C).

### Increased NK cell-mediated apoptosis and reduced size of IFN-γ + TNF-α-treated spheroids

To assess NK cell-functions in the 3D cultures, BC spheroids from the MDA-MB-231 and MCF-7 cell lines were treated with IFN-γ + TNF-α and co-cultured with NK cells.

**Fig. 7:**
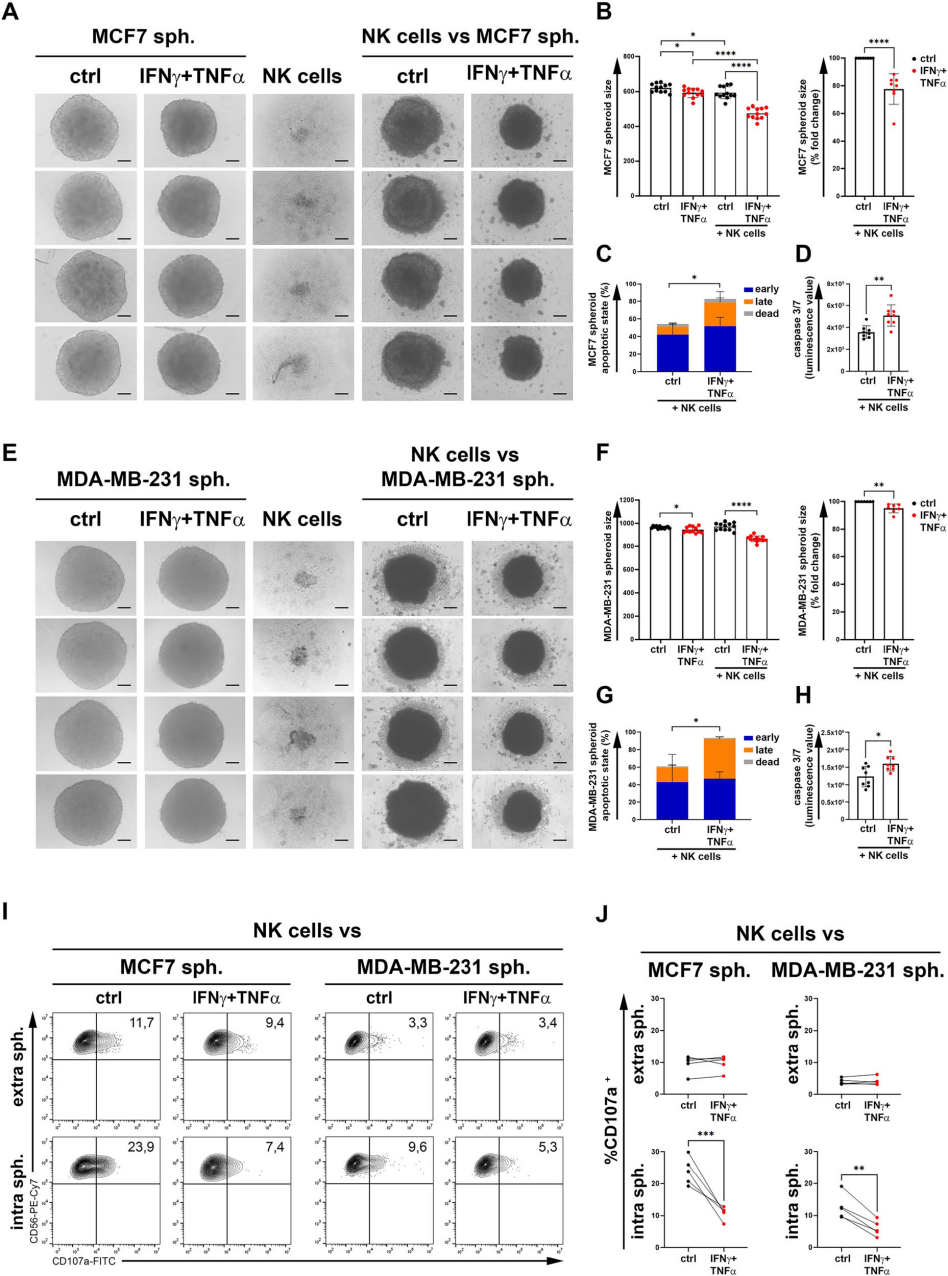
Treatment with IFN-γ + TNF-α makes BC spheroids more susceptible to NK cell-mediated apoptosis. **A** MCF-7 and **E** MDA-MB-231 BC spheroids were either untreated (ctrl) or treated for 24 h with low, non-toxic doses of IFN-γ + TNF-α (IFN-γ + TNF-α) and added of medium or NK cells for additional 24 h. Then, images of the spheroids were acquired by optic microscope (10X magnification). Images of four representative spheroids for each indicated condition, from a representative experiment, are shown. The diameter of the spheroid was measured with ImageJ. A summary of twelve MCF-7 (**B**, left) and MDA-MB-231 (**F**, left) spheroids from a representative experiment is shown. The average size of twelve spheroids of MCF-7 (B, right) and MDA-MB-231 (**F**, right) from eight independent experiments, normalized against controls, was reported as the percentage of fold change. **C** MCF-7 and **G** MDA-MB-231 spheroids, untreated (ctrl) or treated with IFN-γ + TNF-α (IFN-γ + TNF-α) and co-cultured with NK cells were evaluated for apoptotic state. A summary of four independent experiments evaluating the percentage of spheroids in the early (Annexin V^+^ 7-AAD^-^, blue), late (Annexin V^+^ 7-AAD^+^, orange) and dead (Annexin V^-^ 7-AAD^+^, gray) phases of apoptosis is reported. **D** MCF-7 and **H** MDA-MB-231 spheroids, untreated (ctrl) or treated with IFN-γ + TNF-α (IFN-γ + TNF-α) and co-cultured with NK cells were evaluated for the activation of caspase 3/7 by Glomax assay. Luminescence values of caspase 3/7 for each condition were normalized by subtracting values obtained with spheroids alone and those obtained with NK cells alone. A representative experiment of eight spheroids for each condition, out of three performed, is reported. The degranulation of extra and intra spheroids NK cells were evaluated by flow cytometry. **I** A representative experiment of five performed is shown. The percentage of CD56^+^CD107a^+^ was reported in each plot. **J** The summary of five independent experiments is reported. Mean ± SD; *p < 0.05, **p < 0.01; ****p < 0.0001; *p* value, two tailed unpaired Student-*t* test.

Treatment with cytokines alone significantly reduced the growth of both BC spheroids at 48 h, (Fig. 7A, B, E, F), while maintaining the apoptotic state unchanged (Supplementary Fig S5). Co-culture with NK cells induced a significant and stronger reduction in the size of IFN-γ + TNF-α-treated BC spheroids compared to untreated controls (Fig. 7A, B, E, F). The NK cell-mediated size reduction following cytokine treatment was more evident in MCF-7 spheroids, with a size decrease of more than 20% (Fig. 7B, left) versus a decrease of only about 5% in MDA-MB-231 spheroids (Fig. 7F left) compared to untreated spheroids, thus confirming the greater resistance to drug treatment of MDA-MB-231 compared to MCF-7 cells.

To evaluate whether the reduced size in BC spheroids was due to NK cell-function, we assessed the apoptotic state of BC spheroids treated with IFN-γ + TNF-α and co-cultured with NK cells. NK cell-mediated apoptosis of both IFN-γ + TNF-α-treated BC spheroids was significantly increased in the ‘early’, ‘late’ and “dead” states compared to control spheroids (Fig. 7C, G). Furthermore, the significant increase in NK cell-mediated apoptosis of spheroids following cytokine-treatment was confirmed by the caspase 3/7 assay (Fig. 7D, H), thus suggesting an activation of the NK cell-mediated apoptosis by the upregulation of FAS and TRAIL-R2 on IFN-γ + TNF-α-treated BC spheroids, as reported above (Fig. 6).

Furthermore, we evaluated the degranulation of NK cells, assessed by CD107a expression, co-cultured with untreated and IFN-γ + TNF-α-treated spheroids obtained by MCF-7 or MDA-MB-231 cell lines. The degranulation of NK cells infiltrating IFN-γ + TNF-α-treated BC spheroids (intra spheroids) was significantly reduced compared to that of NK cells infiltrating untreated spheroids. In contrast, the degranulation of non-infiltrating NK cells (extra spheroids) in untreated spheroids was comparable to that of non-infiltrating NK cells in IFN-γ + TNF-α-treated BC spheroids (Fig. 7I, J). Of note, the degranulation of infiltrated NK cells was higher than that of non-infiltrated NK cells in untreated BC spheroids (Mean ± SD, in MCF7 spheroids 23,9 ± 4,2 vs 9.5 ± 2.8; in MDA-MB-231 spheroids 12.6 ± 3.8 vs 4.0 ± 0.9), further confirming that intra spheroids NK cells are generally more activated than those remaining outside the spheroids. The significant decrease in degranulation of NK cells infiltrating IFN-γ + TNF-α-treated BC spheroids compared to untreated spheroids was associated with the significant upregulation of MHC-I assessed in IFN-γ + TNF-α-treated BC spheroids compared to untreated spheroids (Fig. 6), thus responsible for negative signals outweighing activating ones [32]. Therefore, these results indicate that the increased in NK cell-mediated killing of IFN-γ + TNF-α-treated BC spheroids was attributable to an increase in NK cell-mediated apoptosis rather that their ability to degranulate.

Overall, these data indicate that treatment with IFN-γ + TNF-α made BC spheroids significantly more accessible to NK cell-infiltration and susceptible to NK cell-mediated apoptosis. Accordingly, these findings suggest that the combined treatment with these cytokines, used at low, non-toxic doses, can enhance the efficacy of NK cell-based immunotherapy of BC.

## Discussion

Cytokines have historically been defined as one of the main family of immunomodulatory molecules [33]. Although the safety of their use in clinical settings has been widely debated and their use has been reduced over time, they remain effective molecules, highly competitive with other immunomodulatory drugs [34–38]. Therefore, the adoption of cytokines in the clinical management of cancer warrants a new consideration.

This study investigated the effects of the cytokines IFN-γ and TNF-α, used at low, non-toxic doses, on the susceptibility of 2D BC cell cultures and 3D BC spheroids to NK cell-mediated anti-tumor activity. The efficacy of low doses of IFN-γ and TNF-α have been reported in different tumor contexts [12, 15] and validated in some clinical settings [39–42]. We here provide evidence on the immunomodulatory effect of the combined treatment with low doses of IFN-γ + TNF-α, which significantly enhances NK cell-infiltration into 3D BC spheroids and NK cell-mediated killing of BC cells, on the one hand by increasing the expression of death receptors such as FAS and TRAIL-R2 and the adhesion molecule ICAM-1 on BC cells cultured in both 2D and 3D conditions and, on the other hand, by increasing the expression of activating receptors on NK cells infiltrating BC spheroids.

The upregulation of death receptors was associated with increased NK cell-mediated apoptosis of BC cells, as validated by neutralizing experiments and in agreement with our previous results obtained in a pediatric tumor model [15]. The increased expression of FAS and TRAIL-R2 should facilitate the extrinsic apoptotic pathway, thus enhancing the NK cell-mediated cytotoxicity of BC cells [43–45].

In addition, the increased expression of ICAM-1 promoted the enhanced formation of NK cell-BC cell conjugates, consistently with previous findings showing that ICAM-1 plays a critical role in supporting NK cell-functions in the context of pediatric tumors [46]. The increased conjugate formation between NK cells and BC cells following combined treatment with IFN-γ + TNF-α was correlated with enhanced interaction between NK cells and tumor cells and an increased apoptotic state of the latter, resulting in reduced spheroid size. This result is consistent with previous reports showing that IFN-γ and TNF-α can increase the susceptibility of leukemic tumor cells to NK cell-mediated lysis through the upregulation of ICAM-1 [14, 46], the expression of which has been shown to be crucial for supporting chimeric antigen receptor (CAR)-mediated NK-cell therapies [47]. Of note, low expression of ICAM-1 in TNBC patients has been associated with a poor treatment response and a worse prognosis, resulting in immune evasion mechanisms [48].

However, the expression of ligands recognized by NKARs, such as NKG2D and DNAM-1, remained largely unaffected or reduced by cytokine-treatment, according with our previous results in neuroblastoma cells [15]. This indicates that the modulation of NK cell-function by IFN-γ + TNF-α-treated BC cells might primarily rely on apoptotic pathway activation and increased expression of adhesion molecules than on the recognition of activating ligands expressed on BC cells.

In the 3D BC cultures, the infiltration of NK cells into the spheroids, also previously reported in other models [49, 50], was enhanced following IFN-γ + TNF-α+treatment, and was associated with increased expression of activating receptors (NKG2D, DNAM-1, NKp30, and NKp46) on spheroid-infiltrating NK cells compared to non-infiltrating NK cells. This finding underlines the importance of the TME in modulating NK cell-responses [11] and highlights that IFN-γ + TNF-α+treatment may enhance NK cell-infiltration in solid tumors. Interestingly, the upregulation of activating receptors was paralleled by the increase of NK cell-exhaustion markers (PD-1 and CTLA-4), particularly in MDA-MB-231 (TNBC) spheroids. This suggests that, while cytokine-treatment promotes NK cell-activation, it may also simultaneously induce their exhaustion, which could limit the efficacy of NK cell-mediated tumor clearance in certain contexts, particularly in more aggressive tumor types such as TNBC. NK cell-exhaustion is a well-documented hallmark in cancer [28], where the prolonged activation of NK cells within the TME leads to the upregulation of inhibitory receptors which impair their cytotoxic function [11, 27, 51].

In addition, our findings indicate that treatment with IFN-γ + TNF-α had a more significant effect on MCF-7 (luminal-A subtype) than on MDA-MB-231 (TNBC) spheroids, when considering both the NK cell-mediated apoptosis and reduction in spheroid size. This is consistent with the clinical observation that TNBCs are generally more resistant to multiple treatments [52], including immune-based therapies, due to their immune evasion mechanisms as loss of tumor-specific antigens, absence of antigen presentation, and aberrant activation of signaling pathways leading to an immunosuppressive phenotype within the TME [53]. The significant response observed in MDA-MB-231 spheroids suggests that immunomodulatory therapies, involving the combined use of low doses of IFN-γ + TNF-α, may also be effective in TNBCs, although a greater response was obtained in MCF-7 cells, due to their more favorable immune profile. This finding highlights the importance of considering tumor heterogeneity when developing immune-based therapies, as different BC subtypes may respond differently to the same treatment.

## Conclusion

In conclusion, this study highlights the potential of using IFN-γ and TNF-α as adjuvants to enhance NK cell-mediated antitumor activity in BC. In fact, treatment with low, non-toxic doses of these cytokines promoted increased NK cell infiltration into the BC spheroids and significantly enhanced NK cell-mediated apoptosis. Cytokine treatment may improve the efficacy of NK cell-based immunotherapies by facilitating the interactions between NK cells and tumor cells through the upregulation of ICAM-1 and enhancing the NK cell-mediated apoptosis of tumor cells through the upregulation of death receptors such as FAS and TRAIL-R2. However, the simultaneous induction of NK cell exhaustion markers, particularly in more aggressive tumor types such as TNBC, raises concerns about the long-term efficacy of this approach. Tumor heterogeneity plays a critical role in therapeutic response, as demonstrated by the more robust effect of cytokine treatment in MCF-7 (luminal-A) spheroids compared to MDA-MB-231 (TNBC) spheroids. These results highlight the need for personalized strategies when considering immunomodulatory therapies, taking into account the unique immune microenvironment and tumor characteristics. Therefore, while cytokines such as IFN-γ and TNF-α offer promise, their use in the clinical settings requires careful optimization considering the tumor subtype and the balance between NK cell-activation and exhaustion.

## Supplementary information

Supplementary Figure S1

Supplementary Figure S2

Supplementary Figure S3

Supplementary Figure S4

Supplementary Figure S5

Supplementary Figure S6

Supplementary figure legends

## Data Availability

The datasets generated and/or analyzed during the current study are available from the corresponding author on reasonable request.
