# Correction: Combined IFN-γ and TNF-α treatment enhances the susceptibility of breast cancer cells and spheroids to Natural Killer cell-mediated killing

**DOI:** 10.1038/s41419-025-08381-7

**Published:** 2026-01-21

**Authors:** Francesca Barberini, Riccardo Pietroni, Simone Ielpo, Valeria Lucarini, Daniela Nardozi, Ombretta Melaiu, Monica Benvenuto, Chiara Focaccetti, Camilla Palumbo, Federica Rossin, Doriana Fruci, Daniel Olive, Laura Masuelli, Roberto Bei, Loredana Cifaldi

**Affiliations:** 1 Department of Clinical Sciences and Translational Medicine, University of Rome “Tor Vergata”, Rome, Italy; 2 Department of Experimental Medicine, University of Rome “Sapienza”, Rome, Italy; 3 Department of Biology, University of Rome “Tor Vergata”, Rome, Italy; 4 Bambino Gesù Children’s Hospital, IRCCS, Rome, Italy; 5 Immunity and Cancer Team, Cancer Research Center of Marseille, CRCM, INSERM U1068, CNRS UMR7258, Aix-Marseille University U105, Marseille, France

**Keywords:** Breast cancer, Immune cell death, Tumour immunology, NK cells

Correction to: *Cell Death & Disease* 10.1038/s41419-025-08021-0, published online 16 October 2025

In this article, section “Materials and Methods” typos need to be corrected:

“BC cell lines cultured in 2D were treated with a cytokine cocktail containing 50 μg/mL IFN-γ (285-IF, R&D) and 20 μg/mL TNF-α (210-TA, R&D) for 48 h.”

with this:

“BC cell lines cultured in 2D were treated with a cytokine cocktail containing 50 ng/mL IFN-γ (285-IF, R&D) and 20 ng/mL TNF-α (210-TA, R&D) for 48 h.”

“BC spheroids were either untreated or treated with a low-dose, non-toxic cytokine cocktail, with cytokine concentration previously established by apoptotic dose-response assays (75 μg/mL IFN-γ and 30 μg/mL TNF-α)”

with this:

“BC spheroids were either untreated or treated with a low-dose, non-toxic cytokine cocktail, with cytokine concentration previously established by apoptotic dose-response assays (75 ng/mL IFN-γ and 30 ng/mL TNF-α)”

The original article has been corrected.

