# Supplementary figures and images for: Combined IFN-γ and TNF-α treatment enhances the susceptibility of breast cancer cells and spheroids to Natural Killer cell-mediated killing
Source: Cell Death Dis. 2025 Oct 16;16(1):729. doi: 10.1038/s41419-025-08021-0 (PMC12533081; doi:10.1038/s41419-025-08021-0)

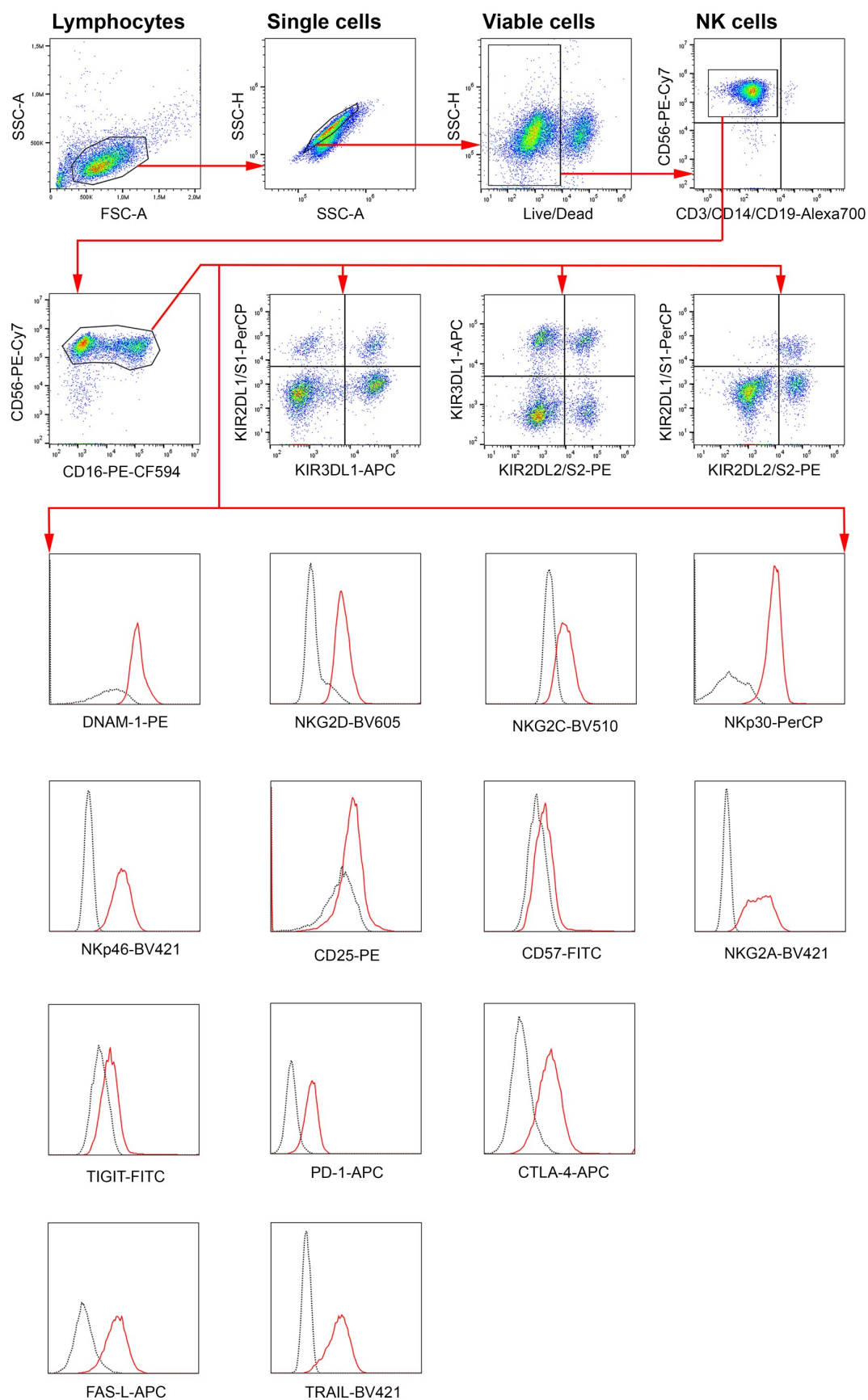

Supplement: Supplementary file 1 — Supplementary Figure S1 [file 41419_2025_8021_MOESM1_ESM.pdf]

**A**

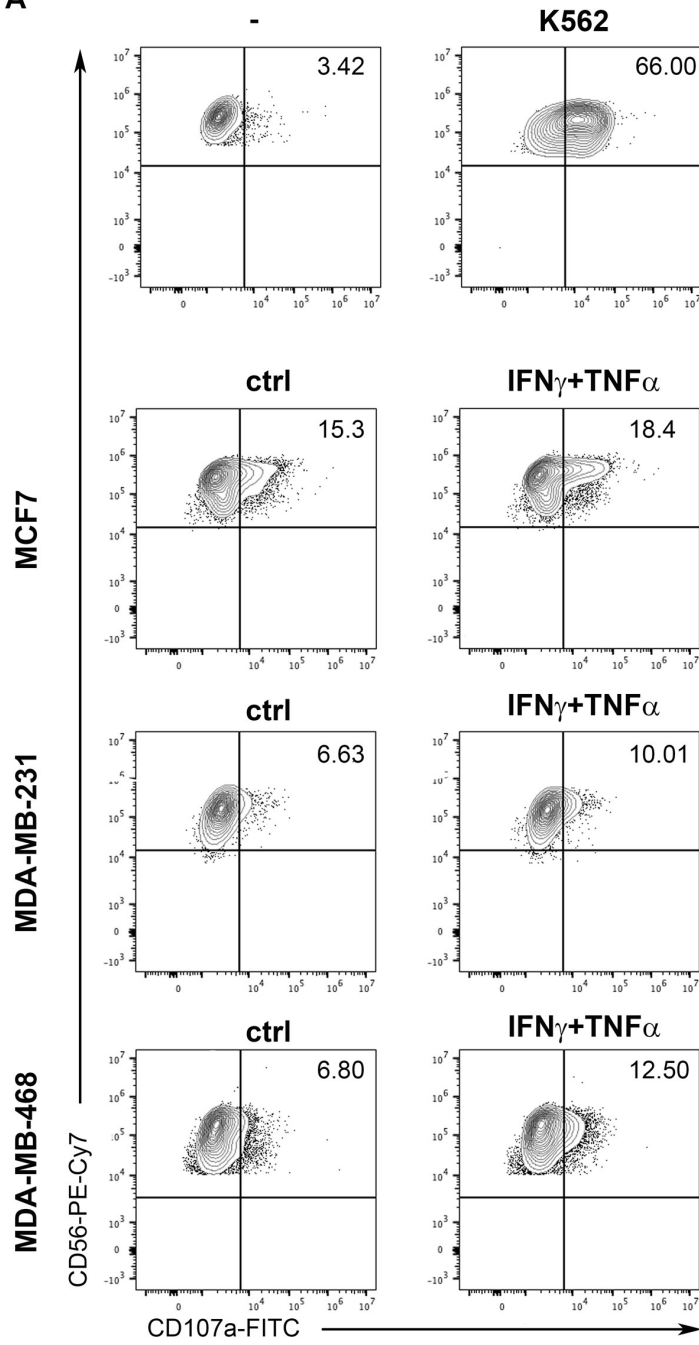

**B**

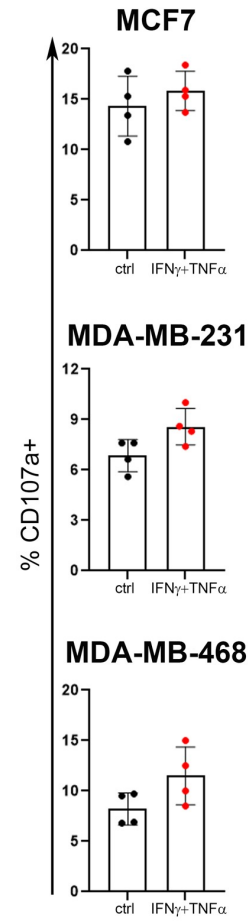

Supplement: Supplementary file 2 — Supplementary Figure S2 [file 41419_2025_8021_MOESM2_ESM.pdf]

NK cells

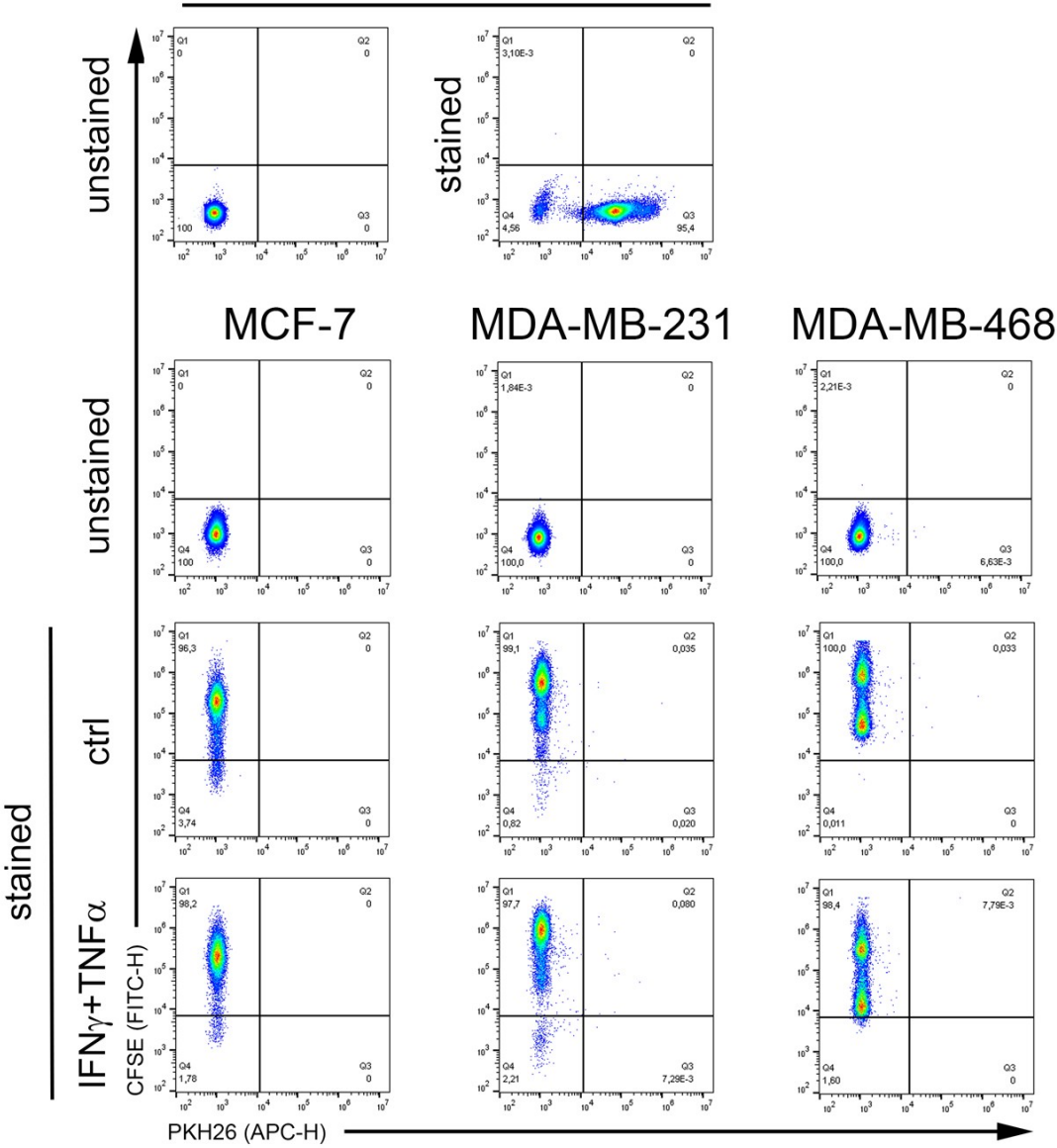

Supplement: Supplementary file 3 — Supplementary Figure S3 [file 41419_2025_8021_MOESM3_ESM.pdf]

## spheroids

---

**MCF-7**

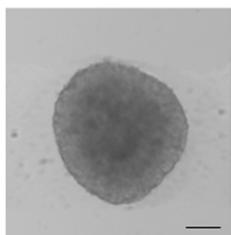

**MDA-MB-231**

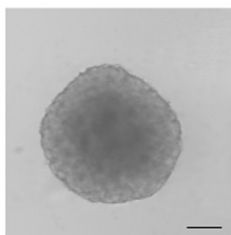

**MDA-MB-468**

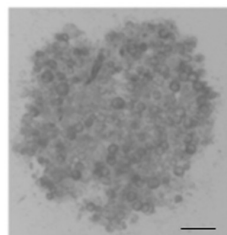

Supplement: Supplementary file 4 — Supplementary Figure S4 [file 41419_2025_8021_MOESM4_ESM.pdf]

## MDA-MB-231 sph.

Top →

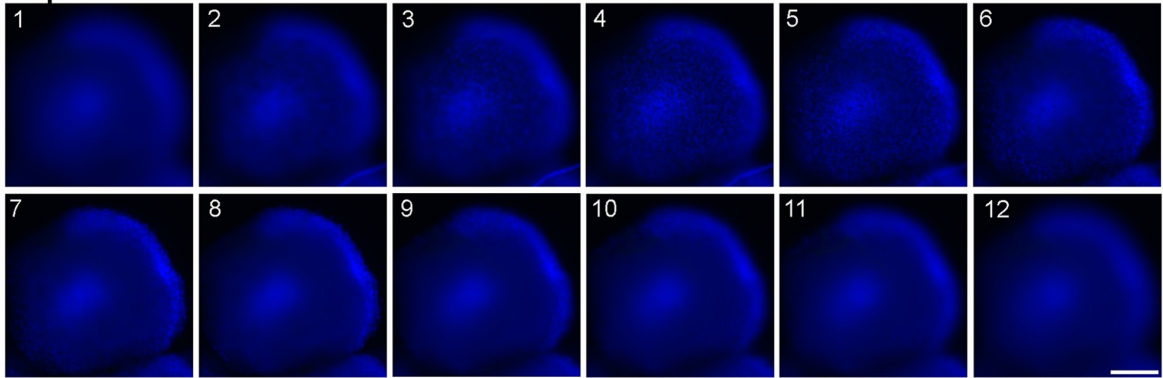

Bottom

Top →

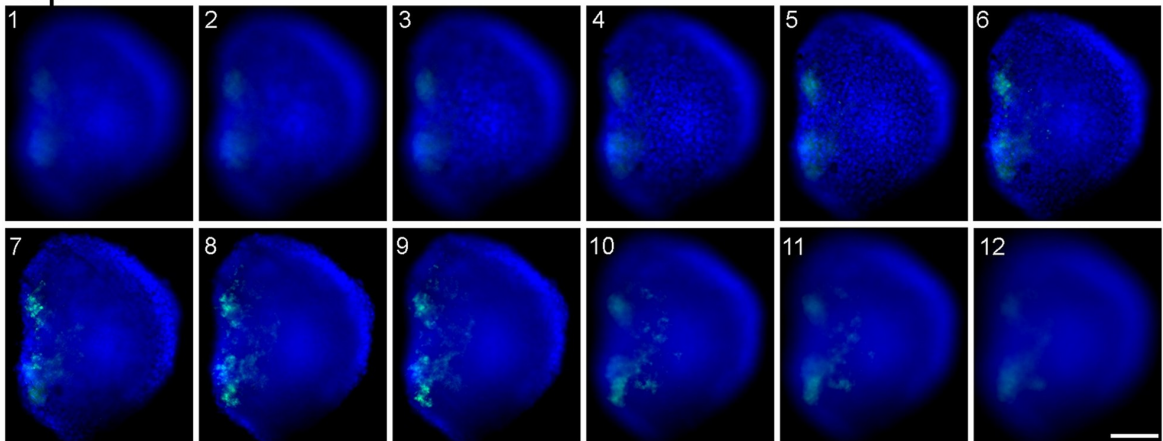

Bottom

NK cells

IFN $\gamma$ +TNF $\alpha$

Top →

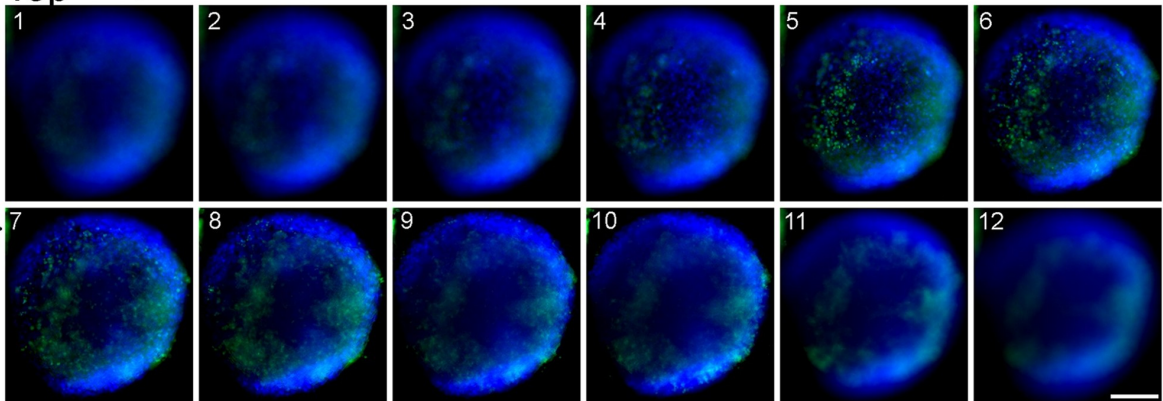

Bottom

Supplement: Supplementary file 5 — Supplementary Figure S5 [file 41419_2025_8021_MOESM5_ESM.pdf]

**MCF-7 spheroids**

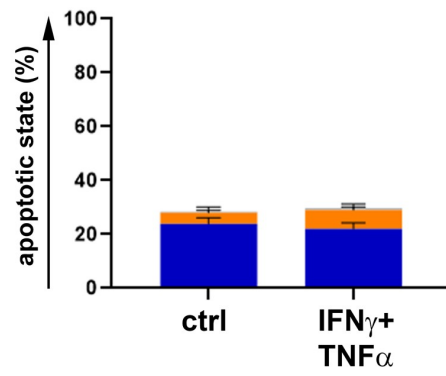

**MDA-MB-231 spheroids**

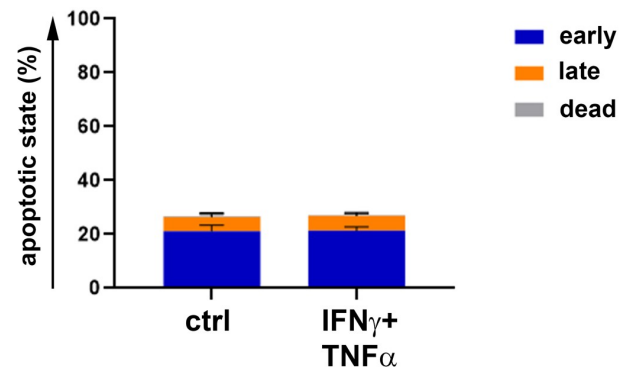

Supplement: Supplementary file 6 — Supplementary Figure S6 [file 41419_2025_8021_MOESM6_ESM.pdf]
