# Supplementary material for: Combined IFN-γ and TNF-α treatment enhances the susceptibility of breast cancer cells and spheroids to Natural Killer cell-mediated killing
Source: Cell Death Dis. 2025 Oct 16;16(1):729. doi: 10.1038/s41419-025-08021-0 (PMC12533081; doi:10.1038/s41419-025-08021-0)
Supplement: Supplementary file 7 — Supplementary figure legends [file 41419_2025_8021_MOESM7_ESM.docx]

**Supplementary Figure S1.** Gating strategy routinely adopted to analyze NK cell phenotype by flow cytometry before using them in experiments. After gating lymphocyte subset (FSC-A vs SSC-A), single cells (SSC-A vs SSC-H), and alive cells (negative for Live/Dead staining), NK cells were identified by gating on CD3^-^ CD14^-^ CD19^-^ CD56^+^ subsets to evaluate in CD56^+^ CD16^+/-^ subset the expression of surface markers, as indicated. In the CD56^+^ CD16^+/-^ subset, the expression of KIRs, activating receptors such as DNAM-1, NKG2D, NKp46, NKp30, and CD25, or immune check point molecules such as PD-1, TIGIT, and CTLA-4, or death molecules such as FAS-L and TRAIL were evaluated. Furthermore, to verify the maturation status of our *in vitro* expanded NK cells, the expression of NKG2A, NKG2C and CD57 in CD56^+^ CD16^+^ subset were evaluated. A representative immunophenotype of the expanded and activated NK cells at 15 days of culture is shown.

**Supplementary Figure S2.** **IFN-γ+TNF-α-treatment of BC cell lines did not affect NK cell degranulation.** MCF-7, MDA-MB-231 and MDA-MB-468 BC cell lines were either untreated or treated for 24 hours with low, non-toxic doses of IFN-γ+TNF-α and then used as target cells in NK cell degranulation assays. K562 target cells were used as positive control. (A) A representative experiment of the four performed is shown. The percentage of CD56^+^CD107a^+^ was reported in each plot. (B) The summary of four independent experiments is reported. Mean + SD; two tailed unpaired Student-*t* test.

**Supplementary Figure S3.** **CFSE^+^ BC cell lines and PKH26^+^ NK cells for conjugation assay.** As controls for the conjugation assay, BC cell lines, either untreated (ctrl) or treated with IFN-γ+TNF-α (IFN-γ+TNF-α) as described above, were left unstained or were stained with CFSE. A representative plot of untreated/unstained, untreated/stained and treated/stained for each BC cell line was reported. NK cells were left unstained or stained with PKH26. A representative plot of unstained and stained NK cells was reported.

**Supplementary Figure S4.** **BC spheroid formation.** MCF-7, MDA-MB-231 and MDA-MB-468 BC cell lines were seeded in low-adherent plates for 24 hours to obtain spheroids. MCF-7 and MDA-MB-231 formed spheroids, while the MDA-MB-468 cell line did not produce spheroids. A representative image of spheroids for each BC cell line acquired under a light microscope (10X magnification) was shown.

**Supplementary Figure S5. Z-stack slide gallery.** Single slide (1-12), from top to bottom, images from z-stack of a representative MDA-MB-231 spheroid alone and untreated (ctrl) or IFNγ+TNFα-treated both co-cultured with NK cells pre-stained with CFSE (green). DAPI was used for nuclear staining (blue). Bars correspond to 100 µm.

**Supplementary Figure S6.** **Treatment with IFN-γ and TNF-α did not affect the apoptotic state of BC spheroids.** MCF-7, MDA-MB-231 spheroids were untreated (ctrl) or treated with low, non-toxic doses of IFN-γ+TNF-α (IFN-γ+TNF-α), as described above, and evaluated for apoptotic state. A summary of four independent experiments evaluating the percentage of spheroid cells in the early (Annexin V^+^ 7-AAD^-^, blue), late (Annexin V^+^ 7-AAD^+^, orange) and dead (Annexin V^-^ 7-AAD^+^, gray) phase of apoptosis is reported.
